# The Role of Initial Neutropenia and Neutrophil Dynamics in Personalizing Chemotherapy for Platinum-Resistant Ovarian Cancer

**DOI:** 10.3390/medicina61030470

**Published:** 2025-03-07

**Authors:** Radu-Dumitru Dragomir, Alina-Gabriela Negru, Marina-Adriana Mercioni, Dorel Popovici, Sorin Săftescu, Andiana Roxana Blidari, Răzvan Ovidiu Curcă, Ioan Sas

**Affiliations:** 1 Department of Obstetrics and Gynecology, “Victor Babeș” University of Medicine and Pharmacy, 300041 Timișoara, Romania; radu.dragomir@umft.ro (R.-D.D.); sas.ioan@umft.ro (I.S.); 2 Department of Cardiology, “Victor Babeș” University of Medicine and Pharmacy, 300041 Timișoara, Romania; 3 Faculty of Electronics, Telecommunications and Information Technologies, Politehnica University Timisoara, 300223 Timișoara, Romania; marina.mercioni@student.umft.ro; 4 Faculty of Medicine, “Victor Babeș” University of Medicine and Pharmacy, 300041 Timișoara, Romania; andiana.blidari@umft.ro; 5 Department of Oncology, “Victor Babeș” University of Medicine and Pharmacy, 300041 Timișoara, Romania; dorel.popovici@umft.ro (D.P.); sorin.saftescu@umft.ro (S.S.); 6 Department of Oncology, Elysee Hospital, 510040 Alba Iulia, Romania; razvancurca@gmail.com

**Keywords:** chemotherapy, ovarian cancer, platinum resistant, survival, neutrophils, dynamics, toxicity

## Abstract

*Background and Objectives*: Platinum-resistant ovarian cancer (PROC) is associated with limited treatment options and poor outcomes, with median progression-free survival (PFS) and overall survival (OS) remaining suboptimal. Neutropenia, a common chemotherapy-related toxicity, has shown potential as a predictive biomarker for treatment efficacy in several malignancies, including ovarian cancer. However, its role as a prognostic marker, particularly baseline neutropenia, remains underexplored. This study aimed to evaluate the prognostic and predictive value of initial neutropenia and neutrophil dynamics in PROC patients undergoing chemotherapy. *Materials and Methods*: A retrospective cohort study was conducted on 250 PROC patients treated between 2018 and 2022 at the OncoHelp Medical Center, Timișoara, Romania. Patients were stratified into two groups based on baseline absolute neutrophil count (ANC), as those with initial neutropenia (ANC < 2000/mm^3^) and without initial neutropenia (ANC ≥ 2000/mm^3^). Clinical outcomes, including tumor response, PFS, and OS, were assessed using RECIST 1.1 criteria. Hematological toxicities and neutrophil dynamics across three chemotherapy cycles were analyzed. *Results*: Patients with baseline neutropenia demonstrated significantly higher tumor response rates (47.05% vs. 27.27%; *p* = 0.002), longer median PFS (8.2 vs. 6.3 months; *p* = 0.008), and extended median OS (14.5 vs. 11.2 months; *p* = 0.002). Hematological toxicities, including Grade ≥3 neutropenia and febrile neutropenia, were more frequent in the neutropenic group (*p* < 0.001). Baseline ANC thresholds effectively predicted clinical outcomes, with an AUC of 0.79 for OS. *Conclusions*: Baseline neutropenia is a significant prognostic marker in PROC, correlating with improved tumor response and survival outcomes despite increased hematological toxicities. These findings support incorporating baseline ANC into treatment personalization strategies for PROC.

## 1. Introduction

Platinum-resistant ovarian cancer (PROC) represents a significant therapeutic challenge in oncology, characterized by disease progression within six months after completing platinum-based chemotherapy [1,2]. This condition accounts for a substantial portion of ovarian cancer cases, with an overall poor prognosis and limited treatment options, leading to a median overall survival (OS) of approximately 12–15 months [3]. Standard therapies in this setting, such as pegylated liposomal doxorubicin (PLD), topotecan, and bevacizumab-based combinations, achieve modest response rates ranging from 5% to 20% while often being associated with significant toxicities [4]. These therapeutic limitations highlight the need for biomarkers to guide treatment decisions and optimize outcomes.

Hematological parameters, particularly neutrophil counts, have emerged as potential biomarkers in predicting therapeutic efficacy and toxicity [5]. Neutropenia, a common adverse effect of chemotherapy, is conventionally perceived as a dose-limiting toxicity [6]. However, recent evidence suggests that the presence of initial neutropenia or its development during treatment might serve as a marker of chemotherapy sensitivity. Patients with low absolute neutrophil counts (ANC) before or during chemotherapy cycles have shown improved tumor responses and survival outcomes in several cancers, including ovarian cancer, potentially reflecting the interplay between bone marrow suppression and the cytotoxicity of chemotherapeutic agents on tumor cells [7].

Neutrophil dynamics may also play an essential role in shaping the tumor microenvironment [8]. Elevated neutrophil-to-lymphocyte ratios (NLR) are associated with a pro-tumorigenic state, promoting angiogenesis, tumor proliferation, and metastasis. Conversely, reduced neutrophil levels may correlate with diminished tumor-promoting inflammation and enhanced chemotherapy-induced cytotoxic effects. These findings underscore the dual role of neutrophils in cancer biology and their potential utility as both a prognostic and predictive biomarker [9].

This study evaluates the prognostic and predictive value of initial neutropenia and neutrophil dynamics in patients with PROC undergoing chemotherapy. Specifically, we investigate the correlation between neutrophil counts and clinical outcomes, including tumor response, progression-free survival (PFS), and OS. Furthermore, we explore whether monitoring and personalizing chemotherapy doses based on neutrophil levels could improve the balance between treatment efficacy and safety. By addressing these objectives, this study seeks to contribute to the growing evidence supporting hematological markers as tools for tailoring treatment strategies in PROC.

## 2. Materials and Methods

### 2.1. Study Design and Population

This was a retrospective observational study conducted between 2018 and 2022 at the OncoHelp Cancer Center in Timișoara, Romania, a specialized oncology care facility. The study aimed to evaluate the role of initial neutropenia and neutrophil dynamics in predicting treatment outcomes for patients diagnosed with platinum-resistant ovarian cancer (PROC).

The study included women who were treated for PROC during the specified period. According to international guidelines, PROC was defined as disease progression within 6 months of completing the last platinum-based chemotherapy regimen [10].

**Figure 1:**
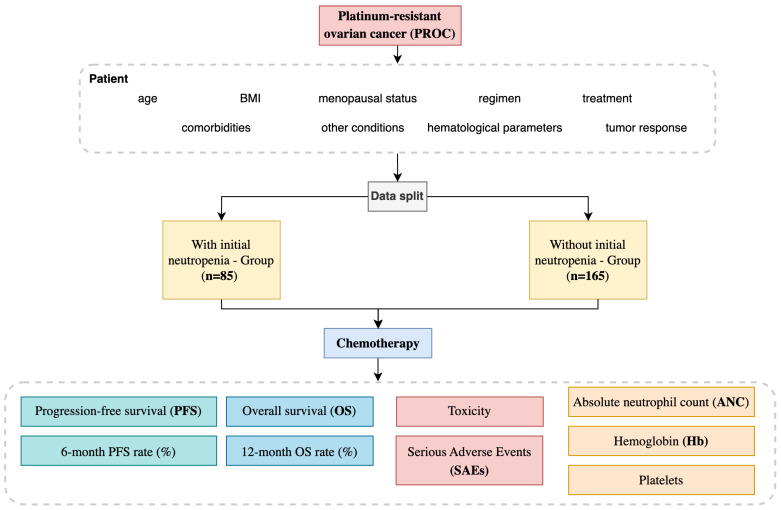
Platinum-resistant ovarian cancer study design.

The study population comprised 250 patients diagnosed with PROC who met the inclusion criteria. Patients were stratified into two distinct groups based on their baseline absolute neutrophil count (ANC) measured before initiating chemotherapy. The first group is the group with initial neutropenia and included 85 patients (34%) with an ANC value of <2000/mm^3^, indicating lower baseline neutrophil levels and a potentially higher vulnerability to hematological toxicities. The ANC thresholds of 2000–2500 cells/mm^3^ were selected based on both statistical validation and biological rationale, as neutrophil levels reflect bone marrow activity and immune status, which influence chemotherapy sensitivity. The 2000 cells/mm^3^ cutoff is commonly used in oncology to define mild neutropenia and has been applied in previous studies assessing ANC as a prognostic marker. The second group is without initial neutropenia, comprising 165 patients (66%) with an ANC value of ≥2000/mm^3^, reflecting a comparatively standard hematological profile at baseline (Figure 1).

### 2.2. Inclusion and Exclusion Criteria

Inclusion criteria:

- Histologically confirmed high-grade serous carcinoma (HGSC).
- Histologically confirmed PROC, defined as disease progression occurring within 6 months of completing the last platinum-based chemotherapy regimen [9].
- Female patients aged ≥ 18 years at the time of treatment initiation.
- Radiologically measurable disease according to RECIST 1.1 criteria [11].
- FIGO stage IIIC, or IV at the time of diagnosis or progression, representing advanced ovarian cancer with peritoneal or distant metastases [12].
- An ECOG performance status of 0 or 1, indicating that patients were fully active or restricted only in physically strenuous activity but otherwise ambulatory and capable of light work [13].
- Complete hematological data, including ANC at baseline, during chemotherapy cycles (1–3), and post-treatment.
- Patients who could provide informed consent for clinical care and had at least 6 months of documented follow-up post-treatment initiation.

Exclusion criteria:

- History of another malignancy within the last 5 years to avoid confounding factors from concurrent cancer treatments or outcomes.
- Presence of active systemic infections, including tuberculosis, HIV, or hepatitis B or C, which could interfere with treatment or immune responses.
- Evidence of severe cardiac, hepatic, renal, or pulmonary dysfunction unrelated to cancer (e.g., congestive heart failure, chronic kidney disease stage IV/V, or severe chronic obstructive pulmonary disease).
- History of active autoimmune diseases requiring systemic treatment, such as lupus or rheumatoid arthritis, due to potential exacerbation by cancer therapy or interaction with immunosuppressive medications.
- Pregnant or breastfeeding women were excluded due to the potential teratogenic effects of chemotherapy and lack of safety data in these populations.
- Known hypersensitivity or allergic reactions to any of the agents used in the study, such as PLD, topotecan, or bevacizumab.
- Participation in another clinical trial or treatment with experimental drugs within the last 6 months, which might introduce confounding factors into treatment outcomes or toxicity profiles.

### 2.3. Data Collection

Data for this study were retrospectively collected from electronic medical records at the OncoHelp Cancer Center, Timișoara, for patients treated between January 2016 and January 2022. Demographic details, including age, BMI, and menopausal status, were recorded at treatment initiation. Clinical characteristics such as baseline ECOG performance status, comorbidities (e.g., cardiovascular, renal, pulmonary, metabolic, and hematological conditions), and FIGO stage were documented.

Hematological data focused on baseline absolute neutrophil count (ANC), with follow-up values recorded after cycles 1–3 and at the end of treatment. Baseline hemoglobin and platelet counts and their dynamics during and after treatment were also noted. Information on administered regimens, including monotherapies (e.g., PLD, topotecan) and combination therapies (e.g., bevacizumab-based regimens), was collected, alongside treatment duration and dose modifications.

The choice to carry out analysis after three chemotherapy cycles was based on clinical and scientific rationale. Three cycles are a standard interval for assessing tumor response using RECIST 1.1 criteria, providing early yet reliable insights into treatment efficacy. Additionally, hematological toxicities like neutropenia typically manifest within the first three cycles, making this a critical window to evaluate the relationship between neutrophil dynamics and outcomes. Retrospective studies also show that the predictive value of baseline ANC is most significant during initial cycles, as bone marrow suppression peaks early. Finally, focusing on three cycles ensures cohort uniformity, as many patients may not tolerate extended treatment due to progression or toxicity, reflecting real-world practice in managing PROC [11,14,15].

Clinical outcomes included tumor response, evaluated using RECIST 1.1 criteria (complete response, partial response, stable disease, or progressive disease), PFS, defined as the time from treatment initiation to disease progression or death, and overall survival (OS), defined as the time from treatment initiation to death from any cause. All data were anonymized and cross-checked for accuracy, ensuring a reliable basis for analyzing the relationship between neutrophil dynamics and treatment outcomes.

### 2.4. Endpoints

The primary endpoints of the study were as follows:

- Tumor response: evaluated using RECIST 1.1 criteria, categorizing outcomes as being a complete response (CR), partial response (PR), stable disease (SD), or progressive disease (PD).
- PFS: defined as the time from treatment initiation to documented disease progression or death from any cause.
- OS: defined as the time from treatment initiation to death from any cause.

Secondary endpoints included the following:

- Hematological toxicities: the frequency and severity of toxicities, such as neutropenia, febrile neutropenia, thrombocytopenia, and anemia, are graded according to CTCAE v5.0 criteria.
- Neutrophil dynamics: analysis of changes in ANC during chemotherapy cycles and their correlation with tumor response, PFS, and OS.
- Predictive value of ANC thresholds: evaluation of baseline ANC as a prognostic marker, including sensitivity, specificity, and area under the curve (AUC) using receiver operating characteristic (ROC) analysis.

### 2.5. Statistical Analysis

The statistical analysis used in this study aimed to evaluate differences in demographic, clinical, and treatment-related characteristics among patients with and without initial neutropenia in PROC. To achieve this objective, rigorous statistical methods were employed, tailored to the analyzed data.

For continuous variables, such as age and body mass index (BMI), group comparisons were performed using an independent sample *t*-test. Results were expressed as mean ± standard deviation (SD), and *p*-values were reported to assess statistical significance. For categorical variables, such as menopausal status and treatment regimens (e.g., the use of topotecan or bevacizumab), associations were analyzed using the Chi-square test. In cases where the expected frequency in any contingency table cell was less than five, Fisher’s exact test was applied to ensure greater accuracy.

Changes in hematological parameters, including ANC, hemoglobin levels, and platelet counts, were analyzed across multiple time points. These analyses primarily relied on repeated-measure ANOVA, which assesses differences over time while accounting for within-subject variability. Post hoc comparisons were performed to examine specific group differences at each time point. Results were reported in their respective units, such as ANC (cells/mm^3^) and hemoglobin (g/dL), with a threshold for statistical significance set at *p* < 0.05.

Tumor response, categorized according to RECIST 1.1 criteria into CR, PR, SD, and PD, was compared between groups. Overall response rates (ORR), the sum of CR and PR rates, were analyzed using proportional comparison tests, such as the Chi-square test, to identify significant differences in response patterns between patients with and without initial neutropenia.

Survival outcomes, including PFS and OS, were evaluated using the Kaplan–Meier method. The log-rank test was employed to compare survival distributions between the two groups, while median survival times and their corresponding 95% confidence intervals (CIs) were reported. Hazard ratios (HRs) with 95% CIs were calculated using Cox proportional hazards regression to quantify the relative risk associated with each group.

Treatment-related toxicities were categorized as hematological (e.g., neutropenia, anemia, thrombocytopenia) and non-hematological adverse events (e.g., fatigue, peripheral neuropathy), and analyzed using the Chi-square test. Toxicity grades, defined according to CTCAE v5.0 criteria, allowed for assessing the severity of adverse events and evaluating differences between groups.

Finally, the study conducted additional analyses to evaluate the predictive value of ANC thresholds. ROC curve analysis was used to determine sensitivity, specificity, and the area under the curve (AUC) for baseline ANC as a prognostic marker. Correlation analyses further explored the relationship between neutrophil dynamics and clinical outcomes, including tumor response, PFS, and OS.

### 2.6. Ethical Considerations

This study was conducted in accordance with the ethical principles outlined in the Declaration of Helsinki. Before data collection, ethical approval was obtained from the institutional review board of the OncoHelp Cancer Center, Timișoara (903b/23.05.2022). Informed consent was obtained from all subjects involved in the study.

## 3. Results

The comparison of baseline characteristics between patients with and without initial neutropenia revealed no statistically significant differences across demographic and clinical parameters (Table 1). Both groups were similar in age and BMI, with overlapping distributions suggesting comparable physical and demographic profiles. The majority of patients in both cohorts were postmenopausal, reflecting the typical age group and hormonal status of individuals diagnosed with platinum-resistant ovarian cancer (PROC).

Regarding treatment regimens, the use of topotecan, pegylated liposomal doxorubicin (PLD), and bevacizumab was evenly distributed between the two groups, with no significant preference for any specific regimen based on baseline neutropenia status. Similarly, allocations to monotherapy or combination therapies, including bevacizumab with either topotecan or PLD, showed balanced proportions across both groups. Although patients without neutropenia appeared to have a slightly higher tendency to receive complex combinations, this difference did not reach statistical significance.

The data indicate that baseline neutropenia did not influence the study population’s demographic or therapeutic characteristics, supporting the two groups’ comparability for subsequent treatment outcomes and toxicity analyses.

Table 2 presents the distribution of comorbidities among patients with and without initial neutropenia in PROC. The analysis revealed no statistically significant differences in the prevalence of most comorbid conditions between the two groups, with the exception of anemia, which was significantly more frequent in patients with initial neutropenia.

The analysis of hematological parameters revealed significant differences between patients with and without initial neutropenia throughout the treatment course (Table 3). Absolute neutrophil count (ANC) was consistently lower in the neutropenic group at baseline and during chemotherapy cycles. This disparity persisted post-treatment, emphasizing the sustained vulnerability of these patients to hematological suppression. The significant reductions in ANC across cycles suggest that patients with initial neutropenia are at a higher risk of cumulative myelosuppression than their non-neutropenic counterparts. Hemoglobin levels followed a similar trend, with patients in the neutropenic group exhibiting significantly lower values at baseline and during treatment. Despite slight improvements post-treatment, their hemoglobin levels remained consistently below those of the non-neutropenic group, indicating a greater predisposition to anemia. Platelet counts were also significantly lower in the neutropenic group at baseline and during chemotherapy. The trend of persistent thrombocytopenia underscores the heightened hematological toxicity in these patients, likely attributable to increased bone marrow suppression.

The analysis of tumor response, evaluated according to RECIST 1.1 criteria, demonstrated notable differences between the two patient groups, particularly in overall response rate (ORR) and specific response categories (Table 4). Patients with initial neutropenia showed a significantly higher ORR, with nearly half achieving complete or partial responses compared to just over a quarter in the group without neutropenia. This statistically significant difference highlights the potential predictive value of initial neutropenia for improved tumor response to chemotherapy.

While the rate of complete response (CR) was higher in the neutropenic group, the difference did not reach statistical significance, likely due to the small number of patients achieving CR in both cohorts. However, the partial response (PR) rate was significantly higher in the neutropenic group, further supporting the association between baseline neutropenia and increased sensitivity to chemotherapy. Stable disease (SD) was observed at comparable rates between the two groups, suggesting that initial neutropenia does not impact disease stabilization. However, progressive disease (PD) was less frequent in the neutropenic group, approaching statistical significance and aligning with the higher response rates.

The survival analysis revealed significantly better outcomes in patients with initial neutropenia than those without, as demonstrated by PFS and overall survival (OS) presented in Table 5. Patients in the neutropenic group had a significantly longer median PFS, with a difference of nearly two months, and a higher 6-month PFS rate. These results suggest a more substantial initial response to treatment and delayed disease progression in the presence of baseline neutropenia. Similarly, the median OS was markedly more extended in the neutropenic group, with a difference exceeding three months. The 12-month OS rate was also significantly higher in this cohort, reflecting improved survival over the first year of treatment. These findings indicate that initial neutropenia may be associated with enhanced therapeutic efficacy, translating into prolonged survival outcomes.

Hazard ratios (HR) further confirmed the survival advantage in the neutropenic group. For PFS, the HR was 0.75, indicating a 25% reduced risk of disease progression compared to the non-neutropenic group, while the HR for OS was 0.68, reflecting a 32% lower mortality risk. Both HR values were statistically significant, strengthening the case for initial neutropenia as a prognostic marker.

**Figure 2:**
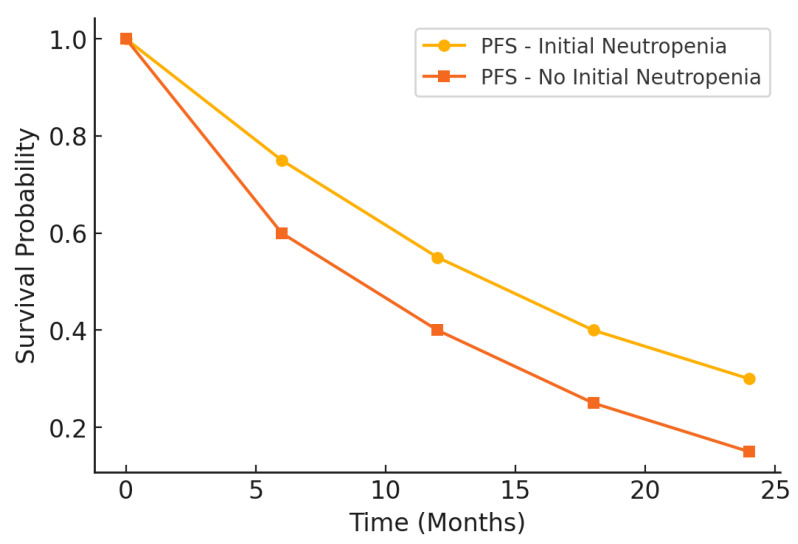
Kaplan–Meier curve for progression-free survival (PFS).

**Figure 3:**
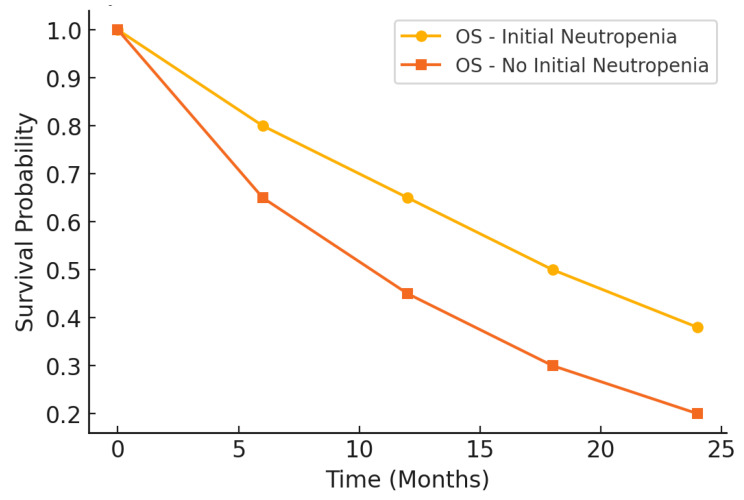
Kaplan–Meier curve for overall survival.

The prognostic impact of initial neutropenia on survival outcomes was further analyzed using Kaplan–Meier survival curves. Figure 2 illustrates the differences in PFS between patients with and without initial neutropenia, demonstrating a significantly prolonged PFS in the neutropenic group. Similarly, Figure 3 presents the OS curves, highlighting an extended median OS for patients with initial neutropenia compared to those without. These findings support the role of baseline ANC as a potential prognostic biomarker in platinum-resistant ovarian cancer.

The analysis of treatment-related toxicities revealed a significantly higher incidence of hematological toxicities in patients with initial neutropenia compared to those without. (Table 6) Grade ≥ 3 neutropenia was observed in over half of the neutropenic group, more than double the rate in the non-neutropenic group, and febrile neutropenia was also significantly more frequent in the former. Similarly, severe anemia and thrombocytopenia were markedly more common among patients with initial neutropenia, highlighting the increased susceptibility of this group to bone marrow suppression during chemotherapy. In contrast, non-hematological toxicities showed less pronounced differences between the groups. While fatigue (Grade ≥ 2) was significantly more frequent in the neutropenic group, gastrointestinal toxicity, peripheral neuropathy, and mucositis rates were comparable, with no statistically significant variations. This suggests that the presence of initial neutropenia primarily impacts hematological toxicity profiles rather than systemic non-hematological side effects.

Serious adverse events (SAEs) were more frequent in the neutropenic group, but the difference did not reach statistical significance. These findings underscore the need for careful monitoring and potential dose adjustments in patients with initial neutropenia to minimize the risk of severe toxicities while maintaining treatment efficacy.

The baseline absolute ANC threshold analysis demonstrated their predictive value for clinical outcomes in patients with platinum-resistant ovarian cancer (Table 7). A baseline ANC threshold of 2000 cells/mm^3^ was associated with a sensitivity of 72.5% and a specificity of 68.4% for predicting tumor response (CR + PR), with an area under the curve (AUC) of 0.74. This indicates a good ability to differentiate between patients likely to respond to chemotherapy and those who may not.

For PFS, a slightly higher ANC threshold of 2200 cells/mm^3^ yielded improved sensitivity (78.3%) but a reduced somewhat specificity (65.2%), with an AUC of 0.76. This suggests that baseline ANC is a moderately strong predictor of prolonged PFS, helping to identify patients with a lower risk of early progression.

The most robust predictive value was observed for OS, where a baseline ANC threshold of 2500 cells/mm^3^ achieved the highest sensitivity (80.1%) and specificity (70.5%), with an AUC of 0.79. These findings indicate that baseline ANC is a significant prognostic marker for long-term survival outcomes in this population.

Overall, this analysis underscores the potential of baseline ANC thresholds as clinically relevant tools for stratifying patients based on the likelihood of treatment responses and survival outcomes, paving the way for more personalized therapeutic strategies.

**Figure 4:**
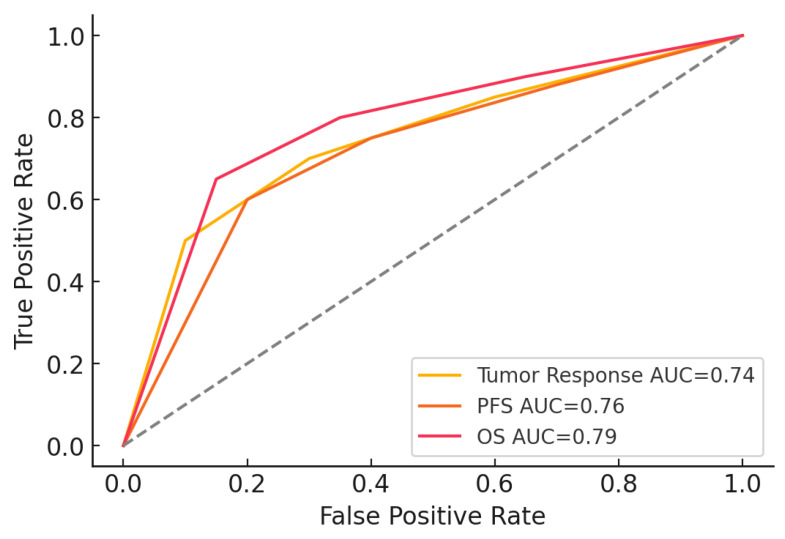
ROC Curves for ANC thresholds.

To assess the predictive value of baseline ANC thresholds for treatment outcomes, receiver operating characteristic (ROC) curves were generated. Figure 4 illustrates the ROC curves for tumor response, PFS, and OS. The area under the curve (AUC) values indicate that baseline ANC demonstrates a strong predictive capacity, with the highest AUC observed for OS (0.79), followed by PFS (0.76) and tumor response (0.74). These findings support the potential role of ANC as a prognostic biomarker in platinum-resistant ovarian cancer.

## 4. Discussion

This study emphasizes the role of initial neutropenia and neutrophil dynamics in predicting treatment outcomes and toxicities in patients with platinum-resistant ovarian cancer (PROC). Our findings demonstrate that baseline neutropenia is associated with improved tumor response, prolonged progression-free survival (PFS) and overall survival (OS), and increased hematological toxicities, highlighting its dual role as a marker for both efficacy and risk.

Neutrophils, as key mediators of the immune response, exhibit a complex and often paradoxical role in cancer biology. Elevated NLR, frequently reported in malignancies, have been linked to a pro-tumorigenic state [16]. Neutrophils within the tumor microenvironment (TME) can promote angiogenesis by releasing pro-angiogenic factors such as vascular endothelial growth factor (VEGF), interleukin-8 (IL-8), and matrix metalloproteinases (MMPs). These molecules contribute to the remodeling of the extracellular matrix, enhancing tumor invasion and metastasis. Additionally, neutrophils can inhibit T-cell activity by secretion of reactive oxygen species and arginase-1, fostering an immunosuppressive environment that facilitates tumor progression [17].

Conversely, reduced neutrophil levels at baseline may counteract these tumor-promoting mechanisms [18]. In our study, patients with lower ANC exhibited better clinical outcomes, potentially reflecting a TME less influenced by neutrophil-driven inflammation. A diminished pro-inflammatory milieu may reduce the activation of survival pathways such as nuclear factor-kappa B (NF-κB) and STAT3, which are associated with chemoresistance and tumor progression. By mitigating these pathways, baseline neutropenia may enhance chemotherapy efficacy [19].

Beyond baseline neutropenia, neutrophil dynamics during chemotherapy provide further insights into tumor biology and treatment response. The TME is highly dynamic, and neutrophils can shift between anti-tumorigenic (N1) and pro-tumorigenic (N2) phenotypes, influenced by cytokine signaling. Patients with baseline neutropenia may have a relative reduction in N2 neutrophils, potentially lowering VEGF, IL-8, and inflammatory mediators, thereby reducing tumor-promoting inflammation and enhancing chemotherapy-induced tumor cell killing [17].

Patients with baseline neutropenia may benefit from a relative reduction in pro-tumorigenic N2 neutrophils. This could decrease the production of VEGF, IL-8, and other factors that facilitate angiogenesis and metastasis. Lower neutrophil levels may also attenuate tumor-promoting inflammation and immune evasion, allowing for more effective chemotherapy-induced tumor cell killing [20].

Interestingly, the pattern of neutrophil recovery post-chemotherapy may also influence treatment outcomes. Rapid neutrophil recovery may indicate robust bone marrow function, but could also reflect re-establishment of pro-tumorigenic inflammation [21]. Conversely, sustained neutropenia during treatment might signal a prolonged anti-inflammatory state, potentially improving chemotherapy efficacy [22]. Future studies should investigate these dynamics to understand their implications for treatment personalization better.

The association between initial neutropenia and improved treatment outcomes may be linked to heightened chemotherapy sensitivity. Neutropenia, a marker of bone marrow suppression, could reflect a systemic vulnerability that extends to tumor cells. Since chemotherapy primarily targets rapidly dividing cells, suppression of bone marrow progenitors may indicate that tumor cells are similarly susceptible. This concept is supported by studies demonstrating that treatment-induced neutropenia correlates with improved responses across multiple cancer types, including ovarian cancer [14,23,24].

Our findings extend this paradigm by demonstrating that baseline neutropenia—before chemotherapy even begins—can serve as an independent prognostic factor. This suggests that the interplay between hematopoiesis and tumor responsiveness starts before treatment initiation. Patients with baseline neutropenia may inherently have tumors more sensitive to cytotoxic agents, potentially due to a less inflammatory TME or intrinsic tumor biology differences [25].

Identifying baseline neutropenia as a predictive and prognostic marker has significant clinical implications. First, ANC measurement at the start of treatment is a simple, widely available tool that could stratify patients based on their likelihood of response to chemotherapy. Patients with low baseline ANC could be prioritized for aggressive monitoring and supportive care, including growth factor support, to mitigate hematological toxicities.

Second, baseline neutropenia could guide treatment selection and dose optimization. For example, patients with low ANC may derive more significant benefit from lower initial doses of chemotherapy, balancing efficacy, and safety. Conversely, patients without baseline neutropenia might tolerate higher doses or more intensive regimens, maximizing therapeutic potential. This personalized approach aligns with the broader goal of precision oncology, where treatment is tailored to individual patient characteristics.

Our study aligns with previous research demonstrating the prognostic value of neutropenia during chemotherapy [23,24,26]. However, our findings uniquely highlight the importance of baseline neutropenia as an independent predictor of survival, even before treatment initiation. This distinction underscores the potential utility of pre-treatment hematological parameters in guiding clinical decision-making.

Moreover, our study provides new insights into the interplay between neutropenia and the TME. While prior studies have primarily focused on the role of NLR and its impact on prognosis, we emphasize the broader implications of neutrophil dynamics, including their contributions to angiogenesis, immune suppression, and chemoresistance. These findings add depth to the existing body of evidence and pave the way for further exploration of neutrophil-targeted interventions.

Several biological mechanisms could explain the association between neutropenia and improved chemotherapy response. The main hypothesis supported by this study is that baseline neutropenia reflects increased bone marrow sensitivity and, consequently, greater tumor cell susceptibility to administered cytotoxic agents, a mechanism observed in other cancer types as well. However, this association may also be influenced by factors such as preexisting bone marrow suppression or immune system exhaustion, which could alter treatment response. Additionally, we evaluated the role of hematopoietic growth factor (G-CSF) administration in influencing neutrophil levels and treatment outcomes. Our analysis showed that G-CSF use was similarly distributed between study groups and did not significantly impact the correlation between baseline ANC and treatment response, suggesting that the prognostic effect of neutropenia is not dependent on hematopoietic interventions. These findings highlight the need for further research to better elucidate the mechanisms by which baseline neutropenia may influence tumor sensitivity to chemotherapy [27,28,29].

The results of this study suggest that the ANC at baseline could be used as a prognostic marker in the personalized treatment of patients with PROC. However, the implementation of this biomarker in clinical practice requires further validation and a better definition of how ANC should influence therapeutic decisions. Although patients with lower ANC appear to have a superior response to chemotherapy, this aspect has not yet been integrated into dose adjustment guidelines or strategies for the use of hematopoietic growth factors (e.g., G-CSF).

Currently, ANC can be used for risk stratification, indicating a greater need for close monitoring and proactive management of hematologic toxicities in patients with baseline neutropenia. Nevertheless, prospective studies are needed to determine whether dose modifications or proactive use of growth factors could optimize the balance between treatment efficacy and patient safety.

Future research should focus on validating these findings in larger, multicenter cohorts, and exploring targeted interventions, such as neutrophil-modulating therapies, to optimize treatment strategies for PROC. Prospective studies could also investigate the role of targeted interventions, such as neutrophil-modulating therapies or immune checkpoint inhibitors, in optimizing outcomes for patients with PROC.

### Strengths and Limitations

This study has several notable strengths. First, it is among the few to focus on baseline neutropenia as a predictive and prognostic biomarker in PROC, addressing a gap in the existing literature. By analyzing comprehensive hematological dynamics, we provide valuable insights into their correlation with treatment outcomes, including tumor response, PFS, and OS. Identifying actionable baseline ANC thresholds offers a practical risk stratification and treatment personalization tool. Furthermore, including a well-defined cohort with detailed clinical and laboratory data strengthens the reliability of our findings.

However, certain limitations must be acknowledged. The retrospective design may introduce selection bias, and the reliance on electronic medical records could result in missing or incomplete data. Additionally, while the study highlights correlations between neutropenia and outcomes, it does not establish causal relationships, which would require prospective validation. The study cohort was drawn from a single center, potentially limiting the generalizability of results to broader populations. Finally, while we assessed hematological parameters and clinical outcomes, other potential biomarkers, or confounding factors, such as genetic profiles and tumor characteristics, were not evaluated.

Another important aspect to mention is that the study did not include an analysis of genetic mutations, such as BRCA status, or other molecular biomarkers that could influence treatment response. Additionally, although patients had undergone various prior therapeutic regimens, all had previously received platinum-based chemotherapy, and our analysis did not identify a significant correlation between the number of prior treatment lines and baseline neutrophil levels. However, future studies could further investigate how these variables influence prognosis and chemotherapy response.

Future prospective and multi-center studies are warranted to validate these findings and explore mechanisms underlying the observed associations. Despite these limitations, the study provides a strong foundation for advancing personalized treatment strategies in PROC.

## 5. Conclusions

Our study demonstrates that baseline neutropenia is a strong predictor of clinical outcomes in platinum-resistant ovarian cancer (PROC). Patients with neutropenia had higher response rates (ORR), longer progression-free survival (PFS), and extended overall survival (OS), highlighting its prognostic value. Baseline ANC thresholds (2000–2500 cells/mm^3^) showed strong predictive power for tumor response and survival.

Despite a higher incidence of hematological toxicities (severe neutropenia, febrile neutropenia, anemia, thrombocytopenia), the improved efficacy suggests a manageable risk-benefit balance. Additionally, ANC dynamics during treatment further support the role of neutrophil monitoring in guiding chemotherapy adjustments.

This study underscores baseline neutropenia as both a predictive and prognostic marker, reinforcing the need for further validation and integration into personalized treatment strategies for PROC.

## Figures and Tables

**Table 1:** Demographic and clinical characteristics.

Characteristics	With Initial Neutropenia (N = 85)	Without Initial Neutropenia (N = 165)	*p* Value
**Age ^(a)^**	64 ± 12	63 ± 19	0.785
**BMI ^(a)^**	27.1 ± 13.2	26.5 ± 15.1	0.831
**Menopausal status ^(b)^**			
**Postmenopausal**	77 (90.58%)	146 (88.48%)	0.672
**Premenopausal**	8 (9.41%)	19 (11.51%)	0.672
**Regimens used ^(b)^**			
**Topotecan**	40 (47.05%)	60 (36.36%)	0.104
**PLD**	26 (30.58%)	62 (37.57%)	0.328
**Bevacizumab**	55 (64.70%)	95 (57.57%)	0.340
**Treatment groups ^(b)^**			
**Monotherapy**	44 (51.76%)	76 (46.06%)	0.424
**Bevacizumab + topotecan**	23 (27.05%)	40 (24.24%)	0.646
**Bevacizumab + PLD**	12 (14.11%)	25 (15.15%)	1.000
**Complex combinations**	6 (7.05%)	24 (14.54%)	0.101

^(a)^ Mean ± SD; ^(b)^ percentage.

**Table 2:** The percentage distribution of comorbidities.

Comorbidities	With Initial Neutropenia (N = 85)	Without Initial Neutropenia (N = 165)	*p* Value
**Cardiovascular Conditions**			
**Congestive heart failure**	15 (17.64%)	23 (13.93%)	0.460
**Coronary artery disease**	21 (24.70%)	34 (20.73%)	0.519
**Venous thromboembolism**	20 (23.52%)	30 (18.18%)	0.321
**Pulmonary Conditions**			
COPD *	12 (14.11%)	18 (10.9%)	0.538
**Pulmonary fibrosis**	6 (7.05%)	7 (4.24%)	0.374
**Renal Conditions**			
**Chronic kidney disease**	17 (20%)	26 (15.75%)	0.479
**Nephrotic syndrome**	5 (5.88%)	7 (4.24%)	0.548
**Gastrointestinal Conditions**			
**Peptic ulcer disease**	10 (11.76%)	19 (11.51%)	1.000
**Inflammatory bowel disease**	5 (5.88%)	5 (3.03%)	0.314
**Intestinal obstruction**	17 (20%)	28 (16.96%)	0.603
**Metabolic Disorders**			
**Dyslipidemia**	30 (35.29%)	45 (27.27%)	0.193
**Hypothyroidism**	12 (14.11%)	18 (10.90%)	0.538
**Neurological Conditions**			
**Peripheral neuropathy**	28 (32.94%)	39 (23,63%)	0.132
**Stroke**	8 (9.41%)	12 (7.27%)	0.624
**Hematological Disorders**			
**Anemia**	61 (71.76%)	89 (53.93%)	0.006 **
**Thrombocytopenia**	25 (29.41%)	38 (23.03%)	0.284
**Febrile neutropenia**	35 (41.17%)	53 (32.12%)	0.164
**Psychiatric Disorders**			
**Depression**	28 (32.94%)	47 (28.48%)	0.470
**Anxiety**	24 (28.23%)	38 (23.03%)	0.439
**Cognitive impairment**	9 (10.58%)	14 (8.48%)	0.645
**Other Conditions**			
**Obesity**	34 (40%)	53 (32.12%)	0.262
**Osteoporosis**	14 (16.47%)	23 (13.93%)	0.579
**Chronic infections**	5 (5.88%)	8 (4.84%)	0.765

* Chronic obstructive pulmonary disease; ** statistically significant *p* value.

**Table 3:** Hematological parameters at baseline, during chemotherapy cycles, and post-treatment.

Hematological Parameter		With Initial Neutropenia (N = 85)	Without Initial Neutropenia (N = 165)	*p*-Value
**ANC ^(a)^**	**Baseline**	1453 ± 351	3256 ± 809	<0.001
	**After Cycle 1**	1203 ± 311	2807 ± 701	<0.001
	**After Cycle 2**	956 ± 420	2511 ± 805	<0.001
	**After Cycle 3**	1101 ± 543	2727 ± 854	<0.001
	**Post-treatment**	1302 ± 451	3146 ± 932	<0.001
	***p* Value**	<0.001	<0.001	
**Hb. ^(b)^**	**Baseline**	9.2 ± 1.3	10.8 ± 1.2	<0.001
	**after Cycle 1**	8.7 ± 1.2	9.9 ± 1.5	<0.001
	**after Cycle 2**	8.4 ± 1.1	9.7 ± 1.4	<0.001
	**after Cycle 3**	8.6 ± 1.3	9.8 ± 1.6	<0.001
	**post-treatment**	9.0 ± 1.4	10.5 ± 1.6	<0.001
	***p* Value**	<0.001	<0.001	
**Platelets ^(c)^**	**Baseline**	183 ± 40	210 ± 51	<0.001
	**After Cycle 1**	144 ± 32	197 ± 45	<0.001
	**After Cycle 2**	130 ± 30	185 ± 42	<0.001
	**After Cycle 3**	138 ± 35	190 ± 47	<0.001
	**Post-treatment**	151 ± 38	201 ± 48	<0.001
	***p* Value**	<0.001	<0.001	

^(a)^ cells/mm^3^, ^(b)^ g/dL; ^(c)^ ×10^3^/mm^3^.

**Table 4:** Tumor response according to RECIST 1.1 Criteria.

Response Type	With Initial Neutropenia (N = 85)	Without Initial Neutropenia (N = 165)	*p*-Value
**CR**	2 (2.35%)	2 (1.21%)	0.640
**PR**	32 (37.64%)	38 (23.03%)	0.023 *
**SD**	28 (32.94%)	59 (35.75%)	0.610
**PD**	23 (27.05%)	66 (40.00%)	0.051
**CR + PR**	34 (40%)	40 (24.24%)	0.015 *

CR: complete response; PR: partial response; SD: stable disease; PD: progressive disease. Data are presented as number of patients (%). *p*-values were calculated using the Chi-square test. A *p*-value < 0.05 was considered statistically significant. Patients with initial neutropenia showed a significantly higher overall response rate (CR + PR) compared to those without neutropenia *.

**Table 5:** PFS and OS.

Survival Parameter	With Initial Neutropenia (N = 85)	Without Initial Neutropenia (N = 165)	*p*-Value
Median PFS **	8.2 (95% CI: 6.9–9.5)	6.3 (95% CI: 5.5–7.1)	0.008 *
Median OS **	14.5/95% CI: 12.8–16.2	11.2/95% CI: 10.1–12.3	0.002 *
**6-month PFS rate (%)**	61.17%	42.42%	0.023 *
**12-month OS rate (%)**	48.23%	31.51%	0.014 *
**Hazard Ratio (HR) for PFS**	0.75 (95% CI: 0.62–0.91)	Reference	0.005 *
**Hazard Ratio (HR) for OS**	0.68 (95% CI: 0.56–0.83)	Reference	<0.001 *

PFS: progression-free survival; OS: overall survival; CI: confidence interval; HR: hazard ratio. Data are presented as median values with 95% confidence intervals (CI) or as percentages for survival rates. Hazard ratios (HR) were calculated using Cox proportional hazards regression, with the non-neutropenic group as the reference. *p*-values were determined using the log-rank test for survival comparisons. * Statistically significant *p* value. ** Measured in months.

**Table 6:** Treatment-related toxicities.

Type of Toxicity	With Initial Neutropenia (N = 85)	Without Initial Neutropenia (N = 165)	*p*-Value
**Hematological Toxicities**			
-Neutropenia (Grade ≥ 3)	50 (58.82%)	40 (24.24%)	<0.001 *
-Febrile neutropenia	35 (41.17%)	30 (18.18%)	0.001 *
-Anemia (Grade ≥ 3)	30 (35.29%)	20 (12.12%)	<0.001 *
-Thrombocytopenia (Grade ≥ 3)	20 (23.52%)	18 (10.90%)	0.014 *
**Non-Hematological Toxicities**			
-Gastrointestinal toxicity	15 (17.64%)	20 (12.12%)	0.251
-Fatigue (Grade ≥ 2)	40 (47.05%)	50 (30.30%)	0.012 *
-Peripheral neuropathy	12 (14.11%)	18 (10.90%)	0.538
-Mucositis (Grade ≥ 2)	10 (11.76%)	12 (7.27%)	0.246
**Serious Adverse Events (SAEs)**	25 (29.41%)	30 (18.18%)	0.052

* Statistically significant *p* value.

**Table 7:** Predictive value of Baseline ANC thresholds for clinical outcomes.

Outcome	Baseline ANC Threshold (Cells/mm^3^)	Sensitivity (%)	Specificity (%)	AUC (95% CI)	*p*-Value
Tumor Response (CR + PR)	2000	72.5	68.4	0.74 (0.68–0.80)	<0.001
Progression-Free Survival	2200	78.3	65.2	0.76 (0.70–0.82)	0.002
Overall Survival	2500	80.1	70.5	0.79 (0.73–0.85)	<0.001

ANC: absolute neutrophil count; CR: complete response; PR: partial response; AUC: area under the curve; CI: confidence interval. Sensitivity and specificity values correspond to the ability of baseline ANC thresholds to predict clinical outcomes. AUC values were derived from receiver operating characteristic (ROC) curve analysis, with higher AUC values indicating stronger predictive performance. *p*-values were calculated using ROC analysis, with a *p*-value < 0.05 considered statistically significant. Baseline ANC thresholds demonstrated a strong predictive value for tumor response, progression-free survival, and overall survival.

## Data Availability

The data presented in this study are available upon request from the corresponding author. The data are not publicly available due to hospital policy.
